# Evaluation of Posterior Segment Structural and Vascular Changes After Gonioscopy-Assisted Transluminal Trabeculotomy

**DOI:** 10.3390/diagnostics16183049

**Published:** 2026-09-20

**Authors:** Umay Güvenç, Serkan Akkaya, Hasan Öncül, Hande Hüsniye Telek, Saniye Doğan, Ayşe Burcu

**Affiliations:** 1 Department of Ophthalmology, Ankara Training and Research Hospital, Hacettepe Mahallesi, Ulucanlar Caddesi, No:89, 06230 Altındağ, Ankara, Türkiye; handetelek@gmail.com (H.H.T.); drsaniyedogan@gmail.com (S.D.); 2 Department of Ophthalmology, Ankara Training and Research Hospital, University of Health Sciences, Hacettepe Mahallesi, Ulucanlar Caddesi, No:89, 06230 Altındağ, Ankara, Türkiye; drsakkaya@hotmail.com (S.A.); anurozler@yahoo.com.tr (A.B.); 3 Department of Ophthalmology, Diyarbakır Gazi Yaşargil Training and Research Hospital, University of Health Sciences, Üçkuyular Mahallesi, Elazığ Yolu Üzeri 10. Km, 21070 Kayapınar, Diyarbakır, Türkiye; hasan.oncul@hotmail.com

**Keywords:** choroidal thickness, gonioscopy-assisted transluminal trabeculotomy, minimally invasive glaucoma surgery, optical coherence tomography angiography, retinal nerve fiber layer thickness, vessel density

## Abstract

**Background/Objectives:** To evaluate longitudinal structural and vascular changes in the posterior segment following gonioscopy-assisted transluminal trabeculotomy (GATT) in primary open-angle glaucoma (POAG). **Methods:** This retrospective observational study included 36 eyes of 36 patients with POAG. Examinations were performed at baseline, at 1 week, and at 1, 3, and 6 months. Optical coherence tomography assessed macular layers and peripapillary retinal nerve fiber layer (RNFL) thickness. Enhanced depth imaging-OCT assessed choroidal thickness (CT). Optical coherence tomography angiography (OCTA) provided superficial and deep macular and peripapillary vessel density (VD). Longitudinal changes in visual acuity (VA), intraocular pressure (IOP), retinal and choroidal structure and microvasculature were evaluated. **Results:** Mean IOP decreased from 19.0 ± 8.3 mmHg to 15.0 ± 3.4 mmHg at 6 months (−20%; *p* = 0.002). VA showed a significant overall time effect (*p* = 0.005), characterized by a transient decline at week 1 and subsequent recovery, with no significant difference between baseline and month 6. RNFL thickness varied numerically across visits, without a significant overall time effect (*p* = 0.504). CT decreased at week 1 and approached baseline values by months 3–6, without a significant overall longitudinal change. No OCTA-derived microvascular parameter remained statistically significant after correction for multiple comparisons. No significant associations were found between IOP reduction and CT or RNFL changes, or between CT and OCTA parameters. **Conclusions:** GATT was associated with moderate IOP reduction, while no statistically significant short-term changes were detected in posterior segment structural and OCTA-derived microvascular parameters during the six-month follow-up.

## 1. Introduction

Gonioscopy-assisted transluminal trabeculotomy (GATT) is an ab interno circumferential trabeculotomy that has emerged as an effective surgical option for lowering intraocular pressure (IOP) in primary open-angle glaucoma (POAG). Its ability to provide sustained IOP reduction with minimal disruption to conjunctival and scleral tissues makes it a favorable alternative to traditional filtering surgeries in selected cases [1,2].

Filtering procedures, such as trabeculectomy (TRAB), are known to induce structural remodeling of the optic disk and macula, as well as alterations in the retinal and peripapillary microvasculature [3,4,5,6,7]. Optical coherence tomography (OCT) and OCT angiography (OCTA) studies have documented postoperative changes in retinal nerve fiber layer (RNFL) thickness, neuroretinal rim metrics, and peripapillary vessel density (ppVD), often correlating with the extent of IOP reduction [8,9,10].

In contrast, the posterior segment effects of angle-based minimally invasive glaucoma surgeries (MIGS), including GATT, remain largely unexplored. Given their more modest and controlled IOP-lowering effect, it is uncertain whether such procedures can meaningfully influence structural and vascular parameters in the optic nerve head (ONH), macula, and choroid. Understanding these changes is important for characterizing the postoperative structural and vascular profile of GATT and may provide further insight into its effects beyond IOP reduction.

To comprehensively characterize these posterior segment responses, complementary structural and vascular imaging parameters were evaluated in the present study. Inner retinal measurements, including macular ganglion cell layer (GCL), inner plexiform layer (IPL), and peripapillary RNFL thickness, were selected as established structural indicators of glaucomatous neuroretinal damage [11]. Central macular thickness (CMT) was included as a structural measure of the overall macular response to glaucoma surgery. Although transient changes in macular thickness have been reported following filtering surgery, the postoperative course of CMT after GATT remains incompletely characterized, particularly in eyes without clinically apparent macular edema [12,13]. Choroidal thickness (CT) was evaluated as a complementary structural marker of postoperative hemodynamic responses, given that the choroid is a highly vascular tissue responsive to alterations in IOP and ocular perfusion following glaucoma surgery [6,14]. Finally, OCTA-derived macular and peripapillary vessel density parameters were included to evaluate postoperative microvascular changes [15,16].

To our knowledge, no previous study has comprehensively assessed both vascular and structural changes in the ONH, macula, and choroid following GATT. Therefore, this study was designed as a longitudinal, descriptive analysis to characterize early structural and vascular changes in the posterior segment following GATT surgery.

## 2. Materials and Methods

This retrospective study included patients who underwent GATT between September 2024 and March 2025. The study protocol was approved by the Institutional Review Board and adhered to the Declaration of Helsinki.

Patients with POAG who underwent GATT because more consistent IOP control and/or reduced reliance on topical therapy was considered clinically necessary to minimize the risk of further glaucomatous progression were included. The primary indication for GATT was classified as uncontrolled glaucoma despite maximum tolerated medical therapy (MTMT), medication non-adherence, medication-related adverse effects or intolerance, or anticipated difficulty maintaining regular long-term follow-up. Each eye was assigned one primary indication based on the principal reason documented in the medical record. MTMT was defined as the greatest number of topical IOP-lowering medication classes that the patient could tolerate while providing additional IOP-lowering benefit.

Eligibility criteria were open iridocorneal angles confirmed on gonioscopy, neuroretinal rim thinning or notching consistent with glaucomatous optic nerve damage, RNFL thinning on OCT, and glaucomatous visual field (VF) defects without other ocular diseases that could explain the findings. VF testing was performed using the Humphrey Field Analyzer 3 with the SITA Standard 24-2 program (model 860; Carl Zeiss Meditec, Dublin, CA, USA). Baseline glaucoma severity was classified according to MD-based Hodapp–Parrish–Anderson criteria [17]. Exclusion criteria included retinal vascular diseases (e.g., diabetic retinopathy, retinal vein occlusion), other ocular conditions affecting the posterior segment, refractive error >±3.00 D spherical or >±2.00 D cylindrical, and postoperative complications such as postoperative hypotony, defined as IOP < 6 mmHg, persistent hyphema lasting ≥1 week, and OCT-confirmed cystoid macular edema. Patients with diabetes mellitus, systemic hypertension, or other systemic vascular disease that could affect ocular perfusion were also excluded.

Each subject underwent a complete ophthalmic examination including best-corrected visual acuity (BCVA), slit-lamp biomicroscopy, gonioscopy, dilated fundus examination, and IOP measurement using Goldmann applanation tonometry (AT 900, Haag-Streit AG, Köniz, Switzerland). Central corneal thickness was measured at baseline, whereas axial length (AL) was measured at baseline and at each postoperative imaging visit using optical biometry (IOLMaster 500; Carl Zeiss Meditec, Jena, Germany).

Structural imaging was performed with spectral-domain OCT (Spectralis; Heidelberg Engineering, Heidelberg, Germany). The macular ganglion cell layer (GCL), inner plexiform layer (IPL), and peripapillary RNFL thickness were obtained using the automated segmentation of the SPECTRALIS Glaucoma Module Premium Edition. Choroidal thickness (CT) was assessed using the enhanced depth imaging mode. Poor-quality scans (signal < 20), misalignment, or segmentation errors were excluded.

Optical coherence tomography angiography (OCTA) was performed with the Avanti RTVue-XR system (Optovue Inc., Fremont, CA, USA) using AngioVue software (version 2018.0.0.14) and the split-spectrum amplitude-decorrelation angiography algorithm. Two volume scans (horizontal and vertical) were acquired to reduce motion artifacts, and vessel density (VD) was calculated automatically using the AngioVue software. Macular scans (6 × 6 mm) were segmented into superficial (SCP) and deep (DCP) capillary plexuses, while optic disk scans (4.5 × 4.5 mm) were used to assess the radial peripapillary capillary plexus.

All OCT and OCTA examinations, including choroidal thickness measurements, were performed between 09:00 and 11:00 a.m. by the same examiner using the same acquisition protocol throughout the study to minimize diurnal variability in choroidal thickness and OCTA-derived vascular measurements. Prior to analysis, all images were reviewed for quality, segmentation errors, and artifacts. Image evaluation was performed independently by two experienced masked graders, and scans with motion artifacts, poor centration, low signal quality, or segmentation errors were excluded. Structural OCT parameters (macular layers and peripapillary RNFL) were extracted directly from the device’s automated segmentation using Anatomic Positioning System-based scan patterns, with visual verification of foveal and Bruch’s membrane opening positions before acceptance. Choroidal thickness was measured independently by two graders using anonymized images, with both graders masked to the visit/timepoint. Measurements were obtained manually at the subfoveal location and at 750 µm nasal and temporal to the fovea. When the intergrader difference was ≤15 µm, the mean of the two measurements was used; differences > 15 µm were reviewed by a third experienced grader, and the final value was determined by consensus [18]. OCTA VD values were generated automatically by the AngioVue software and were recorded without manual modification. Projection artifacts were addressed using the AngioVue system’s built-in removal algorithm, and a signal strength index (SSI) threshold of ≥6 was applied in accordance with the manufacturer’s recommendations.

All GATT procedures were performed by a single experienced glaucoma surgeon (S.A.) using a standard ab interno approach. After a temporal paracentesis, a small goniotomy was created in the nasal trabecular meshwork under gonioscopic view. A 5-0 poly-propylene suture was then advanced circumferentially through Schlemm’s canal for 360°, and both ends were pulled to complete the trabeculotomy. The surgery was completed without external filtering or implants, consistent with the minimally invasive nature of GATT.

Preoperative imaging and IOP measurements were obtained within 15 days before surgery according to the standardized institutional work-up, and patients were informed of the required postoperative follow-up schedule. Postoperative treatment consisted of topical 0.1% dexamethasone and 0.5% moxifloxacin administered four times daily. Moxifloxacin was discontinued after 2 weeks, whereas dexamethasone was gradually tapered thereafter. Routine postoperative clinical examinations, including BCVA, IOP, slit-lamp biomicroscopy, and fundus examination, were performed on days 1 and 2; weeks 1 and 2; and months 1, 2, 3, and 6. OCT and OCTA imaging was performed at baseline and at postoperative week 1 and months 1, 3, and 6. Additional examinations and imaging were performed when clinically indicated, but only data from the five prespecified imaging visits were included in the longitudinal analyses. All antiglaucomatous medications were discontinued at the time of surgery and were not reintroduced during the follow-up period.

During the study period, 58 patients who underwent GATT were screened for eligibility. Of these, 16 were excluded due to coexisting systemic or ocular disease affecting the posterior segment, refractive error outside the predefined range, or incomplete postoperative follow-up. An additional 6 eyes were excluded for postoperative complications: persistent hyphema lasting ≥1 week (*n* = 4), cystoid macular edema (*n* = 1), and hypotony lasting 1 month (*n* = 1). The remaining 36 eyes of 36 patients were included in the final analysis. Only one eye per patient was included; therefore, the cohort consisted of 36 eyes from 36 individual patients, and no bilateral cases were analyzed. In patients eligible for GATT in both eyes, the eye that underwent surgery first was selected; no patient underwent bilateral GATT on the same day.

Because of the retrospective design, no a priori sample size calculation was performed. Instead, a post hoc estimation of the minimum detectable effect size (MDES) was performed to evaluate the sensitivity of the available sample for detecting overall longitudinal changes in the repeated-measures design. Under a repeated-measures within-subject framework, assuming a two-sided alpha level of 0.05, 80% statistical power, a within-subject correlation of 0.5, and a conservative lower-bound nonsphericity correction, the available sample of 36 eyes was sufficient to detect a minimum overall time effect of approximately Cohen’s f = 0.28. Therefore, the available sample size was sufficient to detect moderate longitudinal effects, but may have been insufficient to detect small OCT/OCTA-derived changes.

To facilitate the clinical interpretation of the study sensitivity, we additionally estimated the minimum detectable within-subject differences (MDDs) for representative OCT and OCTA parameters. MDDs were estimated using the standard deviation of paired change scores (SDΔ), assuming a two-sided α of 0.05 and 80% statistical power:

$$MDD=\left(t_{1-\alpha /2,n-1}+z_{1-\beta }\right)\times \frac{SD_{\Delta }}{\sqrt{n}}$$

Statistical analysis was performed using SPSS (version 23.0, IBM Corp., Armonk, NY, USA). Continuous variables were expressed as mean ± standard deviation (SD) or median and interquartile range (IQR), based on distribution assessed via the Shapiro–Wilk test. To address multiplicity, outcome variables were grouped into clinically and anatomically distinct domains, including VA, IOP, retinal structural OCT parameters (CMT, GCL, IPL, RNFL), ONH parameters, CT measurements, macular OCTA metrics, and peripapillary OCTA metrics. Statistical analyses were conducted separately within each domain, with Bonferroni correction applied for post hoc pairwise comparisons. To further control for multiple testing across OCTA parameters, false discovery rate (FDR) adjustment was performed using the Benjamini–Hochberg procedure. Longitudinal changes in IOP, BCVA, retinal layer thicknesses, CT, and OCTA metrics were analyzed using repeated-measures ANOVA (RM-ANOVA), with Greenhouse–Geisser correction applied when sphericity assumptions were violated. The Friedman test was used for non-parametric repeated measures. Post hoc comparisons were conducted using adjusted procedures appropriate to data distribution (including Wilcoxon signed-rank tests for non-parametric data such as VA). For longitudinal analyses with unbalanced repeated measurements, sensitivity analyses were performed using linear mixed-effects models with subject-level random intercepts. To evaluate whether baseline glaucoma severity influenced longitudinal structural, choroidal, and OCTA-derived outcomes, additional linear mixed-effects sensitivity analyses were performed including glaucoma severity category (mild, moderate, severe), time, and the time × severity interaction as fixed effects, with subject-level random intercepts. The primary parameter of interest was the time × severity interaction, which was used to assess whether longitudinal changes differed according to baseline glaucoma severity. Effect sizes were reported as Cohen’s dz for parametric pairwise comparisons, Kendall’s W for non-parametric repeated measures, and partial eta squared (η^2^) for RM-ANOVA. Associations between changes in IOP and CT or RNFL thickness, as well as between CT and OCTA-derived parameters, were assessed using Pearson’s or Spearman’s coefficients, depending on data distribution. A *p*-value < 0.05 was considered statistically significant for overall analyses, while post hoc significance was interpreted using domain-specific adjusted thresholds, with FDR-adjusted *p*-values additionally considered for OCTA parameters where applicable. Mean differences are presented with corresponding 95% confidence intervals (CIs).

## 3. Results

A total of 36 eyes from 36 patients were included (mean age: 66.8 ± 7.0 years; 21 females, 15 males).

The primary indication for GATT was uncontrolled glaucoma despite MTMT in 12 eyes (33.3%), medication non-adherence in nine (25.0%), medication-related adverse effects or intolerance in seven (19.4%), and anticipated difficulty maintaining regular follow-up in eight (22.2%). Uncontrolled glaucoma despite MTMT was the primary indication in one mild, five moderate, and six severe eyes. The preoperative topical medication burden was evaluable in 27 eyes; actual medication use could not be reliably determined in the nine eyes with medication non-adherence. Among these 27 eyes, the mean number of topical IOP-lowering pharmacological classes was 2.52 ± 0.58; 15 eyes (55.6%) were receiving three classes, 11 (40.7%) were receiving two classes, and one (3.7%) was receiving one class (Supplementary Table S1).

No patient had prior laser trabeculoplasty or incisional glaucoma surgery. Lens status was phakic in 15 eyes (41.7%) and pseudophakic in 21 eyes (58.3%); no eye underwent cataract surgery during follow-up, and none developed cataract requiring surgical intervention. Mean central corneal thickness was 536.3 ± 31.0 µm, and mean AL was 23.33 ± 0.65 (range 22.17–24.52) mm. AL showed no significant longitudinal change, measuring 23.29 ± 0.64 mm at week 1, 23.26 ± 0.76 mm at month 1, 23.31 ± 0.76 mm at month 3, and 23.25 ± 0.77 mm at month 6 (Friedman test, χ^2^(4) = 4.67, *p* = 0.323; Kendall’s W = 0.03).

Preoperative VF indices showed a mean visual field index (VFI) of 77.9 ± 25.3, mean deviation (MD) of −8.1 ± 6.9 dB, and pattern standard deviation (PSD) of 5.3 ± 3.3 dB. According to the MD-based Hodapp–Parrish–Anderson classification [17], 17 eyes (47.2%) were classified as mild glaucoma, nine (25.0%) as moderate glaucoma, and 10 (27.8%) as severe glaucoma. To evaluate whether baseline glaucoma severity influenced longitudinal outcomes, sensitivity analyses were performed using linear mixed-effects models including glaucoma severity and the time × severity interaction. No significant time × severity interaction was observed for any structural, choroidal, or OCTA-derived parameter after FDR correction (Table 1).

Mean IOP decreased significantly from 19.0 ± 8.3 mmHg at baseline to 15.0 ± 3.4 mmHg at month 6 (*p* = 0.002, RM-ANOVA). The mean reduction was 4.04 mmHg (95% CI: 1.5 to 6.6), corresponding to a moderate effect size (Cohen’s dz = 0.60). Bonferroni-adjusted post hoc analysis indicated that IOP was significantly reduced at months 1, 3, and 6 compared to baseline. VA showed a different trend. Median VA declined slightly at week 1 compared to baseline, then improved, reaching 0.00 logMAR by month 6. This change was significant (*p* = 0.005, Friedman test), with a small-to-moderate effect size (Kendall’s W = 0.20). Post hoc analysis confirmed a significant difference between week 1 and all subsequent visits (Table 2).

**Figure 1:**
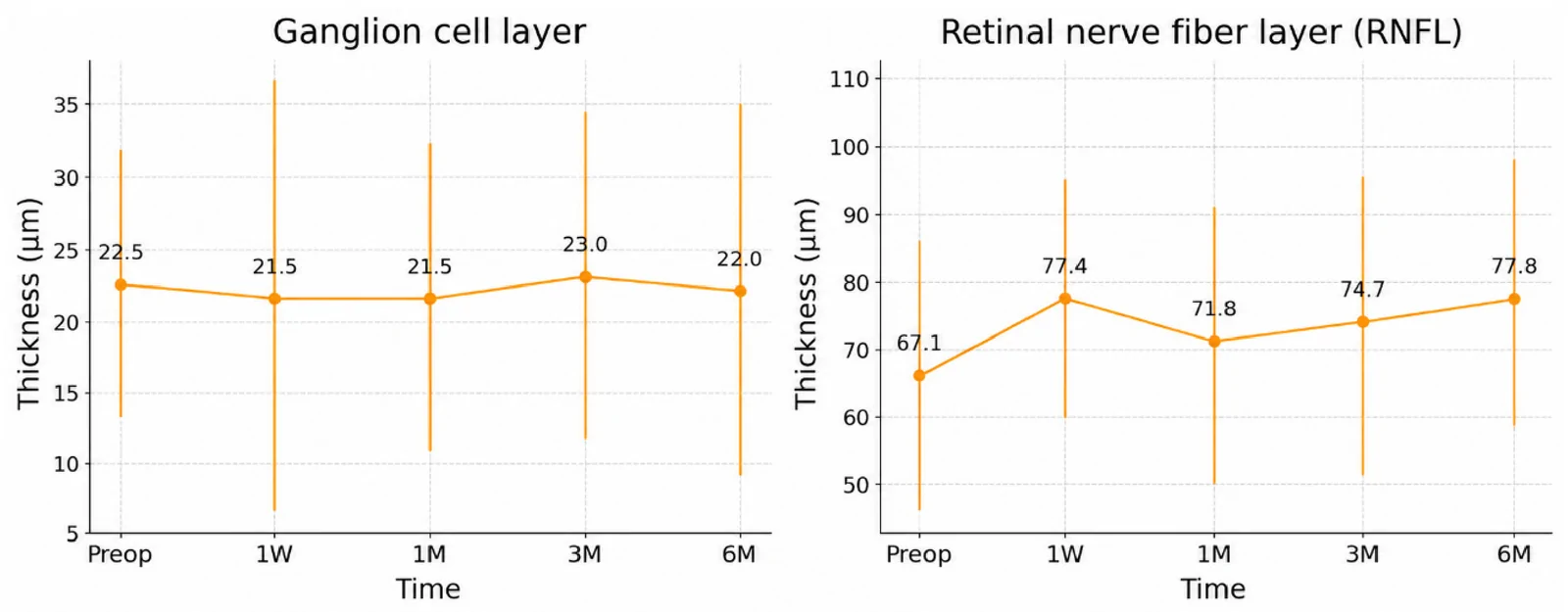
Longitudinal changes in macular ganglion cell layer (GCL) and retinal nerve fiber layer (RNFL) thickness after GATT surgery. Error bar plots (mean ± SD) showing the evolution of the GCL (**left**) and RNFL (**right**) thickness after GATT surgery. RNFL thickness showed a non-significant increase at week 1 followed by a decline at month 1. Numeric labels indicate the mean thickness values at each timepoint.

No significant longitudinal changes were observed in central retinal layers or RNFL thickness during follow-up. CMT, GCL, and IPL showed no significant changes over six months (*p* = 0.190; *p* = 0.936; *p* = 0.765, respectively). RNFL thickness varied numerically across visits, but the overall effect of time was not statistically significant (*p* = 0.504) (Table 3, Figure 1). Effect size analysis demonstrated small effect sizes (η^2^ range: 0.01–0.04) across all retinal parameters, indicating that the magnitude of observed changes was limited.

Given the substantial variability in RNFL measurements, a linear mixed-effects model with a random intercept for subjects was additionally applied. This analysis similarly showed no significant effect of time on RNFL thickness (*p* = 0.092). The marginal R^2^ (R^2^m = 0.06) was low, indicating that time explained only a small proportion of the variance in RNFL measurements, consistent with the repeated-measures ANOVA findings.

Given the clinical relevance of postoperative IOP control in advanced disease, an exploratory analysis was performed in the 10 eyes with severe glaucoma. Mean IOP decreased from 21.10 ± 2.77 mmHg at baseline to 17.10 ± 2.98 mmHg at month 6 (Wilcoxon signed-rank test, *p* = 0.008). RNFL thickness showed no significant change over the same period (49.70 ± 15.02 µm at baseline vs. 53.30 ± 14.36 µm at month 6; Wilcoxon signed-rank test, *p* = 0.016; rank-biserial r = 0.73), which did not reach the Bonferroni-adjusted threshold. All 10 eyes remained free of IOP-lowering medication and did not require additional glaucoma surgery during the six-month follow-up.

ONH parameters, including cup-to-disk (C/D) area, rim area, disk area, and cup volume, remained unchanged throughout follow-up. All parameters demonstrated small effect sizes (η^2^ range: 0.02–0.04), suggesting a limited magnitude of change over time (Table 4).

**Figure 2:**
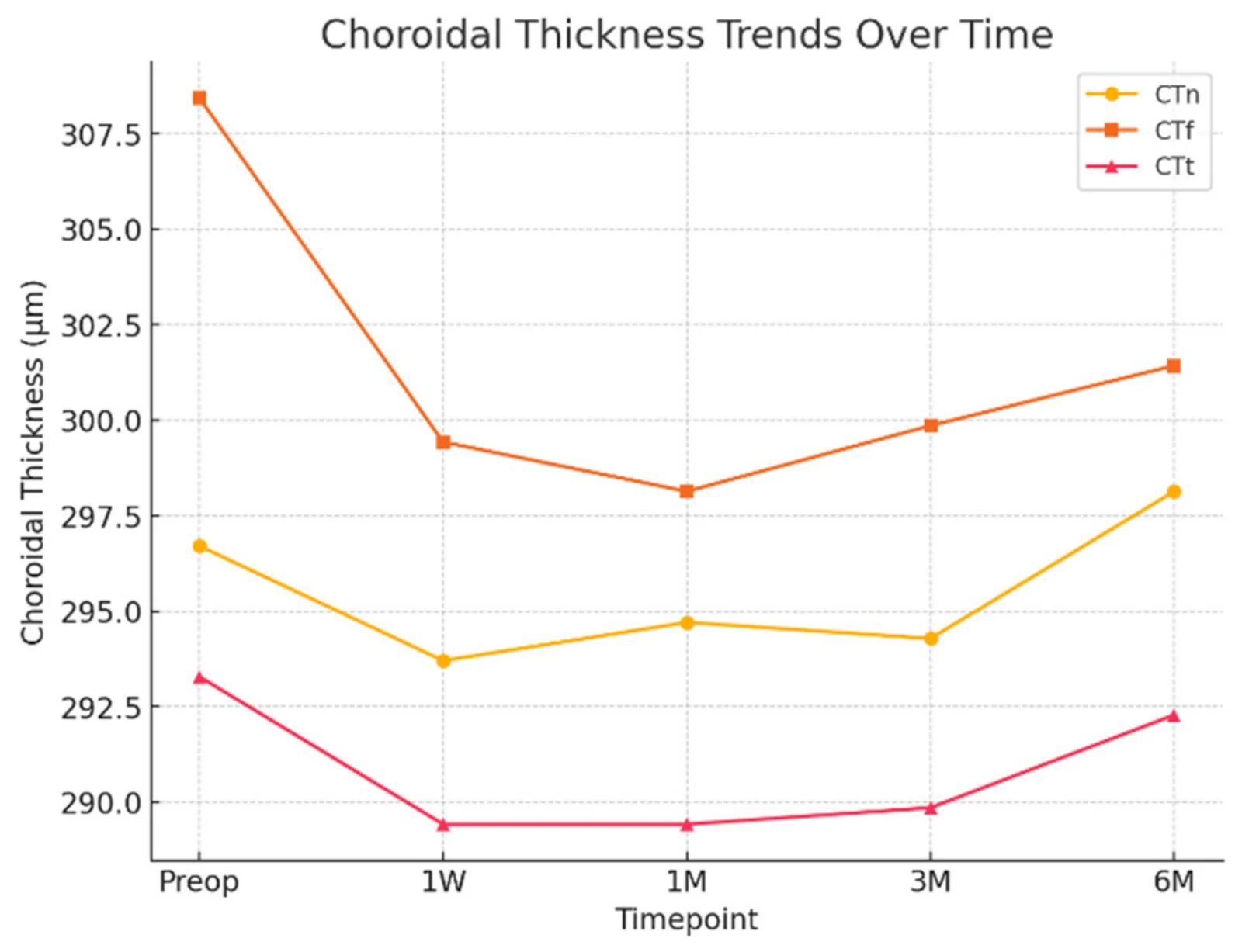
Longitudinal analysis of choroidal thickness trends over time. Line plots (mean ± SD) demonstrate the longitudinal changes in nasal (CTn), subfoveal (CTf), and temporal (CTt) choroidal thickness across the 6-month follow-up period. Although no statistically significant differences were detected (RM-ANOVA, all *p* > 0.05), a transient postoperative reduction, particularly at week 1, was followed by partial recovery toward baseline values by months 3 and 6.

Choroidal thickness (CT) measurements in the nasal (CTn), subfoveal (CTf), and temporal (CTt) regions showed no significant changes over time. CT values were lower at week 1 and approached baseline levels by months 3 and 6. A non-significant tendency toward change was observed in CTn (*p* = 0.059), with a small-to-moderate effect size (η^2^ = 0.06) (Table 5, Figure 2).

Signal strength index (SSI) was recorded at each available OCTA imaging visit to evaluate longitudinal differences in scan quality. The mean ± SD (median [Q1–Q3]) SSI was 7.28 ± 1.03 (7 [6–8]) at baseline, 7.11 ± 0.75 (7 [7–8]) at week 1, 7.53 ± 0.81 (7.5 [7–8]) at month 1, and 7.25 ± 0.87 (7 [7–8]) at month 3. SSI did not differ significantly across visits (Friedman test, χ^2^(3) = 6.68, *p* = 0.083; Kendall’s W = 0.06), and no pairwise comparison reached statistical significance.

**Figure 3:**
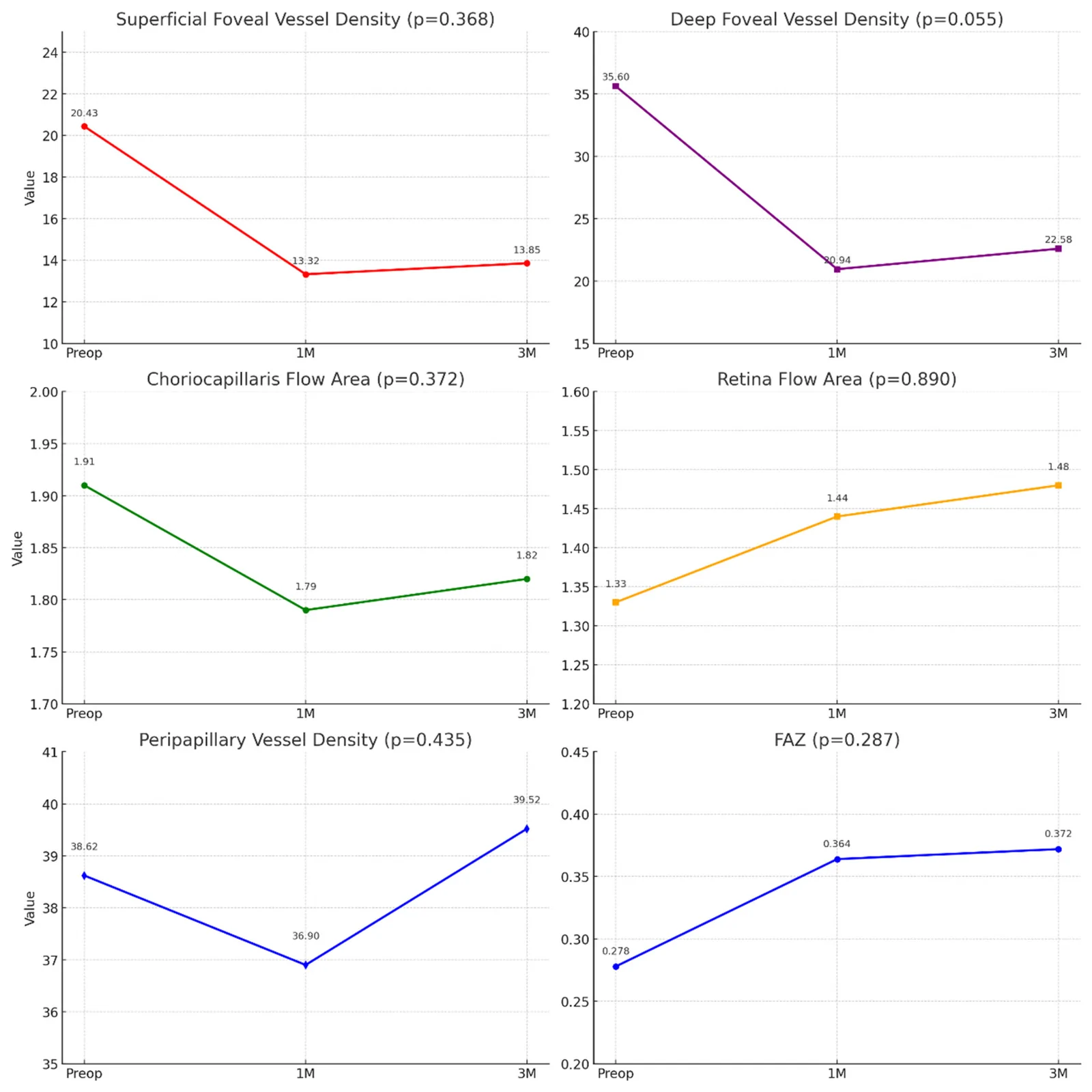
Longitudinal analysis of optical coherence tomography angiography (OCTA) parameters. Line plots (mean ± SD) illustrate changes in superficial and deep foveal vessel density, choriocapillaris and retinal flow area, peripapillary vessel density, and foveal avascular zone (FAZ) from baseline to 3 months after surgery.

OCTA findings demonstrated no consistent statistically significant postoperative changes across vascular layers after FDR adjustment. Post hoc analysis with Bonferroni correction showed a significant reduction at month 1, followed by partial recovery by month 3; however, these differences did not remain statistically significant after multiple comparisons. Whole-image ppVD also showed a significant overall effect of time, with post hoc analysis confirming a reduction at month 1 compared to baseline; this finding similarly did not retain statistical significance after FDR correction. Deep foveal VD did not demonstrate a statistically significant change over time (*p* = 0.055). No significant changes were observed in choriocapillaris or retinal flow areas (Table 6, Figure 3). FDR-adjusted *p*-values are provided in Table 6.

Pearson’s correlation analysis revealed no statistically significant associations between choroidal thickness parameters (CTn, CTf, CTt) and OCTA metrics, including FAZ, superficial/deep foveal VD, choriocapillaris flow, retinal flow area, or peripapillary VD (all *p* > 0.05). A full summary of correlation coefficients is provided in Supplementary Table S2.

**Figure 4:**
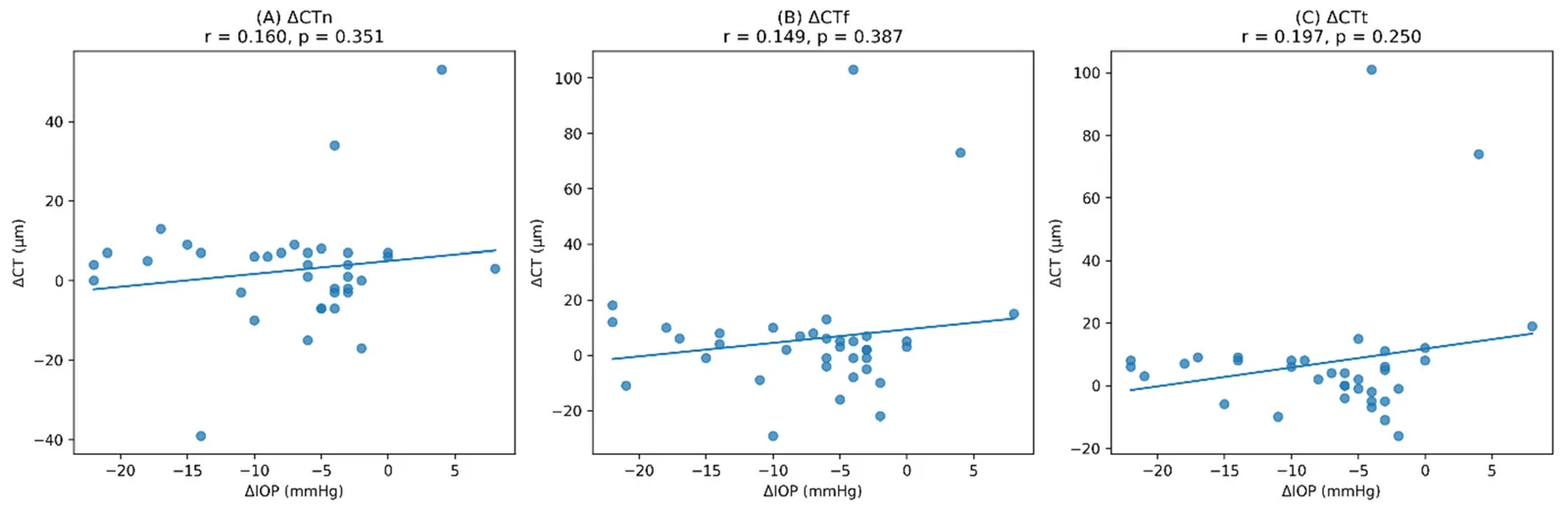
Correlation analysis between the changes in intraocular pressure (ΔIOP) and choroidal thickness. Scatterplots showing the relationships between changes in intraocular pressure (ΔIOP) and nasal (ΔCTn), subfoveal (ΔCTf), and temporal (ΔCTt) choroidal thickness. Linear regression lines are shown for visualization of associations. None of the correlations reached statistical significance (all *p* > 0.05).

Pearson’s correlation analysis was performed using change scores defined as the difference between baseline and month 6 values. No significant associations were observed between IOP reduction and changes in CT in any region (ΔCTn: r = 0.160, *p* = 0.351; ΔCTf: r = 0.149, *p* = 0.387; ΔCTt: r = 0.197, *p* = 0.250) (Figure 4).

Similarly, Spearman’s analysis showed no significant associations between IOP reduction and changes in RNFL thickness. Correlation coefficients were weak across global RNFL (ρ = −0.167, *p* = 0.509), superior (ρ = −0.004, *p* = 0.991), and inferior sectors (ρ = −0.348, *p* = 0.359).

## 4. Discussion

To our knowledge, this is the first study to evaluate early postoperative OCT-derived structural and OCTA-derived vascular posterior segment changes following GATT surgery in patients with POAG. The main findings can be summarized as follows: (i) GATT achieved a moderate but significant reduction in IOP over six months; (ii) no significant short-term changes were detected in retinal structural parameters; and (iii) OCTA-derived vascular parameters showed no statistically significant longitudinal changes after correction for multiple testing.

A key concept that may help interpret the absence of significant OCTA changes in our study is “lower pressure range autoregulation.” When baseline IOP is relatively low or moderately controlled, ocular autoregulatory mechanisms may remain effective within a compensatory range, thereby potentially limiting measurable changes in VD following modest postoperative IOP reduction [10,19]. This concept may be relevant to our cohort, in which baseline IOP was relatively low (19.04 mmHg) and the magnitude of IOP reduction was modest (~20%). Supporting this, Ch’ng et al. [20] and Lommatzsch et al. [10] reported limited vascular changes in cohorts with relatively low baseline IOP (21.0 mmHg) or modest IOP reduction, suggesting that both baseline IOP and the magnitude of reduction may influence the vascular response. However, because OCTA-derived VD does not directly measure ocular blood flow or autoregulatory capacity, preserved autoregulation remains a hypothesis rather than a demonstrated mechanism in the present study.

In contrast, larger IOP reductions, as observed in studies on filtering procedures, may exceed this compensatory capacity and have been associated with more pronounced vascular changes [5,21]. El-Haddad et al. [22] suggested that marked IOP reduction (58–59%) may relieve mechanical compression on the ONH microvasculature, thereby improving perfusion. Similarly, Ellakwa et al. [23] reported substantial IOP reductions (55–60%) and demonstrated a significant association between DCP VD and the degree of IOP reduction. Consistently, Baek et al. [24] identified IOP reduction as the primary determinant of postoperative microvascular improvement, showing an association with decreased choroidal microvascular dropout.

By comparison, MIGS procedures such as GATT are generally associated with modest IOP reduction compared with filtering surgery [25]. Applebaum et al. [26] reported that XEN implantation provides a less invasive surgical option for patients requiring moderate IOP reduction, while Wang et al. [27] observed a 29.2% IOP reduction following a MIGS procedure (trabeculotome tunneling trabeculoplasty), highlighting the relatively modest pressure-lowering effect of such interventions. Consistent with this, Reitemeyer et al. [19] reported a greater magnitude of IOP reduction in the TRAB group compared to XEN at 6 months, which was accompanied by higher macular VD in the TRAB group. Notably, baseline IOP values in that study were relatively modest (17.6 ± 3.8 mmHg for XEN and 21.2 ± 5.4 mmHg for TRAB), and the authors suggested that limited IOP reduction in moderately controlled glaucoma may not be sufficient to induce measurable vascular changes [19].

These findings should also be interpreted in relation to baseline patient characteristics and disease severity. El-Haddad et al. [22] reported significant increases in ppVD as early as one month following TRAB, which they partly attributed to the younger age of their cohort (mean 56 years). They suggested that younger patients may exhibit greater vascular responsiveness due to enhanced microvascular adaptive capacity [22]. In our cohort, in contrast, the mean age was higher (66.8 years), which may have also contributed to a reduced vascular response due to age-related attenuation of microvascular adaptability.

In our study, nearly half of the eyes had mild glaucoma, with an overall mean MD of −8.1 dB. Reitemeyer et al. [19] suggested that baseline disease stage may influence the postoperative vascular response. In our cohort, the sensitivity analysis revealed no significant time × severity interaction for any structural, choroidal, or vascular parameter; however, this should be interpreted with caution, as stratifying the modest sample by glaucoma severity substantially reduced the number of eyes available at each disease stage and limited the power to detect severity-dependent differences. Within the broader literature, disease severity has been variably associated with the postoperative vascular response: Hong et al. [28] reported no significant change in ppVD or macular VD after TRAB in mild-to-moderate glaucoma, with only a numerical decrease, whereas Miraftabi et al. [21], who included patients with more advanced VF loss, observed more pronounced postoperative vascular changes. Taken together, the relatively modest IOP reduction achieved with GATT, along with baseline IOP levels, patient characteristics, and disease stage, may collectively contribute to the absence of significant vascular changes observed in our cohort.

Notably, uncontrolled glaucoma despite MTMT was the primary indication for GATT in six of the 10 eyes with severe glaucoma. In these eyes, GATT was selected as a less invasive, conjunctiva-sparing initial surgical approach within a stepwise treatment strategy, preserving more invasive filtering procedures as subsequent options [1]. The medication-free postoperative course may partly reflect the continuous, adherence-independent IOP-lowering effect of surgery, which may be particularly relevant in patients receiving multidrug regimens, among whom adherence is frequently suboptimal [29]. In addition, the efficacy of chronic topical therapy may decline over time in some drug classes [30], while the incremental IOP-lowering benefit of adding further agents is limited in patients already receiving multiple medications [31]. Beyond its effect on mean IOP, GATT may provide more consistent pressure control; reductions in daytime IOP fluctuation have previously been reported following angle-based MIGS [32]. However, IOP fluctuation was not measured, and the lack of a medically treated control group precludes comparison with medical therapy. These findings support short-term medication-free control, although longer follow-up is needed to determine whether additional treatment will be required in eyes with severe glaucoma.

SCP and DCP vessel density showed an early numerical decline followed by partial recovery, a temporal pattern that was directionally similar to the “delayed response” described by Ch’ng et al. [20], who observed limited early change followed by a later increase in macular vessel density. However, because these changes did not remain statistically significant after correction for multiple comparisons, the observed fluctuations may partly reflect measurement variability rather than a consistent vascular response. SSI did not differ significantly across visits (*p* = 0.083), providing no evidence of a systematic longitudinal change in overall scan quality. However, stable SSI does not exclude other OCTA-specific sources of variability; transient reductions in capillary flow during the early postoperative period may fall below the OCTA detection threshold, potentially leading to underestimation of VD [20,33].

RNFL thickness varied numerically across visits, with an increase at week 1 followed by a decline at month 1; however, no significant overall longitudinal change was detected (*p* = 0.504). Time explained only a small proportion of the variance in RNFL measurements (marginal R^2^ = 0.06), with most variability attributable to between-subject differences. Similar short-term postoperative variations in RNFL thickness have been reported after various intraocular surgeries and have been attributed to transient postoperative inflammatory or physiological responses rather than true structural remodeling [9,20,34,35,36,37]. However, given the non-significant overall time effect and substantial measurement variability, the observed RNFL fluctuations should therefore be interpreted descriptively without attribution to a specific biological mechanism.

Overall, our results suggest that posterior segment structural changes were limited during the short-term follow-up after GATT. In contrast, previous studies on filtering procedures have reported structural changes associated with greater and more abrupt IOP reductions. For example, Vessani et al. [38] reported increased rim area and macular parameters, along with reduced cup-to-disk ratio following TRAB, while Aydin et al. [39] and Ghanem et al. [40] demonstrated significant RNFL thickening associated with marked IOP reduction (~55%). These changes have been interpreted as possible transient mechanical effects of IOP reduction, including tissue decompression and restoration of axoplasmic flow, rather than true neuroretinal gain [39,40].

Macular thickness also showed no significant longitudinal change during the six-month postoperative follow-up. Similar findings have been reported by Nilforushan et al. [12], Demirtaş et al. [3] and Asaoka et al. [41], with no significant postoperative changes observed in retinal layers. Even with profound IOP lowering, macular structural parameters generally remain stable across studies [3,12,41].

Previous studies on filtering procedures have reported postoperative CT thickening, which has been attributed to large IOP reductions, increased ocular perfusion pressure, and postoperative inflammation [6,7,42]. This phenomenon has also been proposed as an early stage of hypotony maculopathy, although its exact mechanism remains uncertain [43,44]. The absence of hypotony in our patients may partly explain the lack of postoperative CT thickening. Instead, we observed a non-significant, transient decrease in CT during the early postoperative period. Previous studies have suggested that, in the setting of modest IOP reduction, early postoperative CT changes may reflect transient hemodynamic adjustments or alterations in aqueous outflow dynamics rather than sustained increases in ocular perfusion pressure, suggesting a dynamic but likely physiological postoperative response [8,45,46,47]. However, because the observed change was not statistically significant and relevant hemodynamic, physiological, and inflammatory variables were not systematically assessed, this mechanistic interpretation remains tentative.

Furthermore, no significant association was found between ΔIOP and ΔCT, suggesting that under conditions of modest IOP reduction, early CT changes may not be directly driven by IOP alone. Consistent with this, Gambini et al. [48] reported no significant association between IOP reduction and CT changes after microshunt implantation, while Luo et al. [47] observed early CT thinning followed by stabilization at 6 months, without a clearly defined relationship to the magnitude of IOP reduction.

We observed a transient decline in VA at one week following GATT, followed by gradual recovery by six months. Similar early recovery patterns have been reported after GATT [1] and may reflect transient hyphema, mild inflammation, or media disturbance associated with surgical manipulation and irrigation; however, the relative contributions of these factors could not be determined in this retrospective cohort. Although median BCVA changed from 0.25 logMAR at baseline to 0.00 logMAR at month 6, this difference was not statistically significant and the overall longitudinal effect size was modest (Kendall’s W = 0.20). The numerical change should therefore not be interpreted as evidence that GATT directly improves VA and may partly reflect normal test–retest variability and postoperative recovery. Supporting the latter possibility, Pillunat et al. [49] reported a postoperative reduction in corneal densitometry, indicating improved corneal transparency, following successful glaucoma surgery in a cohort with a mean preoperative IOP similar to that of the present study. However, because corneal densitometry, endothelial function, and longitudinal refractive changes were not assessed in our cohort, this explanation should be regarded as hypothesis-generating. A change of this magnitude may also be influenced by the expected variability of logMAR VA measurements, particularly in an older glaucoma population such as ours (mean age, 66.8 years). Test–retest variability of VA has been shown to increase with age [50], and a recent systematic review reported pooled limits of agreement of approximately ±0.20 logMAR for distance VA measurements [51]. No eye underwent cataract surgery during follow-up, making lens extraction an unlikely explanation for the observed numerical change.

Our findings should be interpreted within the broader methodological context of OCTA studies in glaucoma surgery, which are commonly characterized by relatively small cohorts, short follow-up durations, and the evaluation of multiple imaging endpoints. For example, Yin et al. [52] reported OCTA changes in 22 eyes with a one-month follow-up using a signal strength threshold of ≥6, while similar studies have included small cohorts (21–34 eyes) with follow-up periods ranging from one to six months [21,22,53]. Prospective studies have also shown comparable designs. Güngör et al. [4] and Ellakwa et al. [23] evaluated 20 and 30 eyes, respectively, over six months using the same OCTA system and similar signal strength criteria, with Ellakwa et al. [23] additionally reporting a priori sample size estimation of 25 eyes. Comparable patterns are also observed in alternative surgical approaches, with Zéboulon et al. [33] evaluating 21 patients undergoing non-penetrating sclerotomy and Reitemeyer et al. [19] comparing XEN implantation and TRAB in small cohorts (32 vs. 13 eyes). Across these studies, the evaluation of multiple OCTA-derived parameters is common, often without uniform adjustment for multiple comparisons, reflecting the exploratory nature of OCTA-based endpoints [4,8,10,14,19,20,21,23,28,33,53]. In our study, additional control for multiplicity using FDR adjustment resulted in the loss of statistical significance for OCTA findings, underscoring the sensitivity of these parameters to analytical approach, and indicating that Bonferroni-based findings should be interpreted with caution in the context of multiple testing. In the context of both our sample size estimation and the existing literature, the study may have been sufficient to detect moderate longitudinal changes. However, small effect sizes—particularly for parameters such as ppVD and DCP metrics—may require larger cohorts, and subtle changes in these measures may not have been fully captured. Therefore, OCTA-derived changes should be interpreted with caution, and future studies with larger sample sizes, more standardized multiplicity control strategies, and stricter signal strength thresholds are warranted.

The potential influence of antiglaucomatous medications on OCTA-derived vascular and structural parameters should be considered when interpreting postoperative findings. Because all medications were discontinued at the time of surgery, the observed changes cannot be attributed to the surgical procedure alone and may reflect the combined effects of surgery and medication withdrawal. The available literature suggests that the vascular effects of topical antiglaucoma medications are complex and time-dependent. Reported changes in OCTA-derived vascular parameters vary according to the medication studied, baseline ocular characteristics, disease severity, magnitude of IOP reduction, and timing of assessment. In a randomized clinical trial, topical timolol produced no significant change in macular vessel density at any retinal location, whereas latanoprostene bunod increased it [54]. Topical carteolol has likewise been reported to increase peripapillary vessel density in some patients while decreasing it in others [55]. A prospective study reported significant increases in optic nerve head, peripapillary, and macular VD one month after initiation of topical latanoprost treatment. Notably, these vascular changes regressed by month 3 despite continued treatment, indicating that they represented transient effects associated with treatment initiation rather than a sustained pharmacological action on the microvasculature [56]. In the present cohort, no OCTA-derived parameter retained statistical significance after correction for multiple comparisons. Therefore, although medication withdrawal remains a potential confounder, its specific contribution to the OCTA findings cannot be determined from the present data.

This constraint is widely recognized across surgical OCTA studies, in which patients are typically imaged under ongoing medical therapy without a washout period [24,26,28]. For instance, Demirtaş et al. [3] and Çiçek et al. [14] did not standardize preoperative medications, interpreting this as representative of real-world clinical conditions, while prospective and comparative studies have similarly acknowledged the inability to fully control for medication-related confounding despite more structured designs [4,19,20,21,22,23]. In this context, our design—without preoperative washout but with uniform postoperative discontinuation—represents a limitation in terms of controlling medication-related effects.

An additional consideration is that, although washout periods have been established for IOP recovery, the time course of OCTA-derived vascular parameters following medication discontinuation remains poorly characterized. Consequently, the potential contribution of medication withdrawal to early follow-up OCTA and choroidal findings cannot be precisely quantified. This limitation extends beyond the present study and reflects an area that remains incompletely understood in the current literature. Accordingly, early follow-up OCTA and choroidal findings should be interpreted with appropriate caution, as the contribution of medication withdrawal cannot be excluded. Future prospective studies incorporating a standardized preoperative washout or a medically treated control group will be required to determine the relative contributions of surgery and medication withdrawal to the observed vascular changes.

The same consideration extends to the primary IOP outcome: because postoperative IOP was likewise measured after complete medication discontinuation, the observed reduction reflects a transition from a medically treated to a medication-free state rather than an isolated surgical effect. However, established washout data indicate that the IOP-lowering effect of topical glaucoma medications, including prostaglandin analogs—the class with the longest duration of action—typically resolves within 4 to 6 weeks of discontinuation [57]. Any residual medication effect is therefore unlikely to have persisted at month 6, although the earlier postoperative measurements may still have been influenced.

This study is limited by its retrospective design, small sample size, and short follow-up, which may reduce statistical power and limit long-term conclusions. A formal numerical target IOP was not consistently documented, precluding assessment against individualized therapeutic targets. Because baseline IOP was measured during prescribed medical therapy and no medication-free preoperative measurement was available, the magnitude of the IOP reduction attributable specifically to GATT could not be isolated. In addition, heterogeneity in the primary indications for surgery—including insufficient control despite MTMT, medication non-adherence or intolerance, and difficulty maintaining regular follow-up—may have contributed to baseline IOP variability and may limit direct comparisons across these clinically distinct subgroups. The absence of a medically treated, non-surgical control group with repeated OCT/OCTA imaging limits our ability to distinguish postoperative stability from normal test–retest variability and temporal fluctuations, while the lack of an alternative surgical group precluded comparison with other procedures. Because eyes with persistent hyphema, hypotony, or cystoid macular edema were excluded, the findings apply to a selected cohort without these complications and should not be extrapolated to complicated postoperative courses. Although the post hoc MDES estimation indicated that the available sample was sufficient to detect moderate longitudinal effects, the study may have been underpowered to detect small OCT/OCTA-derived changes. Likewise, the sensitivity analyses according to baseline glaucoma severity were based on small samples and were not adequately powered to detect severity-specific differences; therefore, the absence of a significant time × severity interaction should not be interpreted as evidence that disease severity does not influence postoperative structural or vascular responses. OCT and OCTA measurements remain susceptible to artifacts, particularly residual projection artifacts in the deep capillary plexus despite built-in correction. Although two graders assessed image quality, formal inter-rater reliability was not evaluated, and the signal strength threshold of ≥6 may have resulted in lower image quality than the higher thresholds used in recent studies. Choroidal thickness measurements were obtained independently by two graders masked to visit order; however, interobserver reproducibility was not quantified, and measurement variability therefore cannot be excluded. Furthermore, blood pressure and hydration status were not systematically recorded, and ocular perfusion pressure and objective inflammatory markers were not assessed; therefore, residual physiological variability and the proposed hemodynamic or inflammatory mechanisms could not be directly evaluated. Finally, given the number of parameters analyzed, there remains a potential risk of type I error, particularly for secondary outcomes. Although multiplicity was addressed using domain-based analysis and FDR adjustment, OCTA findings did not retain statistical significance after correction, underscoring the limited power to detect small effect sizes and the exploratory nature of these results.

To our knowledge, this is the first study to evaluate both structural and vascular posterior segment changes following GATT. While posterior segment responses after filtering surgery have been extensively studied, longitudinal data on combined structural and vascular outcomes after GATT remain limited. The most relevant prior study by Soyugelen et al. [1] reported no significant change in ppVD in 44 eyes with advanced glaucoma, while noting preserved VA. Our findings extend these observations by including a broader range of glaucoma severity, from mild to severe, rather than advanced disease alone, and by assessing both macular and peripapillary vascular parameters. Consistent with their results [1], we likewise observed no significant change in ppVD, and in our cohort, no OCTA-derived vascular parameter remained statistically significant after correction for multiple testing.

From a clinical perspective, in this selected cohort of uncomplicated POAG eyes, no significant short-term OCT or OCTA change was detected following GATT despite a moderate reduction in IOP. Together, these findings provide a clinically relevant description of the early postoperative course after GATT, although the six-month follow-up does not permit conclusions regarding longer-term structural, vascular, or functional outcomes. Further comparative studies with longer follow-up and a medically treated control group are needed to distinguish surgery-related changes from test–retest variability and to characterize the longitudinal vascular response to GATT.

## Figures and Tables

**Table 1:** Baseline glaucoma severity distribution and sensitivity analyses according to glaucoma severity.

**A. Baseline Glaucoma Severity Classification**			
**Severity**	**n (%)**	**MD (dB)**	**RNFL (µm)**
Mild	17 (47.2)	−2.73 ± 2.62	78.2 ± 11.2
Moderate	9 (25.0)	−7.87 ± 1.36	63.3 ± 13.1
Severe	10 (27.8)	−16.66 ± 4.08	51.7 ± 18.9
**B. Linear mixed-effects sensitivity analyses for baseline glaucoma severity**			
**Outcome parameter**		**Time × Severity *p***	**FDR-adjusted *p***
Peripapillary RNFL (µm)		0.189	0.553
Central macula thickness (µm)		0.646	0.718
CTf (µm)		0.277	0.553
Macula SCP VD (%)		0.603	0.718
Foveal SCP VD (%)		0.263	0.553
Macula DCP VD (%)		0.423	0.604
Foveal DCP VD (%)		0.105	0.522
Whole-image ppVD (%)		0.909	0.909
Inside disk ppVD (%)		0.381	0.604
Peripapillary VD (%)		0.059	0.522

Abbreviations: MD = mean deviation; RNFL = retinal nerve fiber layer; CTf = subfoveal choroidal thickness; SCP = superficial capillary plexus; DCP = deep capillary plexus; VD = vessel density; ppVD = peripapillary vessel density. Glaucoma severity was classified according to Hodapp–Parrish–Anderson criteria based on visual field mean deviation (MD). Linear mixed-effects models included time, glaucoma severity, and the time × severity interaction as fixed effects, with subject-level random intercepts. No time × severity interaction remained statistically significant after false discovery rate (FDR) correction.

**Table 2:** Intraocular pressure (IOP) and visual acuity (VA) over time.

Parameter	Pre-Op	Post-Op 1 Week	Post-Op 1 Month	Post-Op 3 Month	Post-Op 6 Month	*p*-Value	Post Hoc Comparisons
IOP (mmHg, mean ± SD)	19.04 ± 8.32	16.00 ± 4.09	15.00 ± 3.81	14.84 ± 3.32	15.00 ± 3.41	0.002 (RM-ANOVA)	Pre-op vs. 1M: *p* = 0.004 Pre-op vs. 3M: *p* = 0.003 Pre-op vs. 6M: *p* = 0.004
VA (logMAR, median [min–max])	0.25 (0.00–1.35)	0.35 (0.10–1.20)	0.10 (0.00–0.20)	0.10 (0.00–0.28)	0.00 (0.00–0.10)	0.005 (Friedman test)	1W vs. 1M: *p* = 0.004 1W vs. 3M: *p* = 0.003 1W vs. 6M: *p* < 0.001

Abbreviations: Pre-op = preoperative; post-op = postoperative; IOP = intraocular pressure; VA = visual acuity; logMAR = logarithm of the minimum angle of resolution; SD = standard deviation; min = minimum; max = maximum; RM-ANOVA = repeated-measures analysis of variance; W = week; M = month; post hoc pairwise comparisons were performed across all timepoints using Bonferroni correction (adjusted significance threshold *p* < 0.005); only statistically significant comparisons are presented. Significant results are shown in bold.

**Table 3:** Changes in macular layers and retinal nerve fiber layer thickness over time.

Layer	Pre-Op	Post-Op 1 Week	Post-Op 1 Month	Post-Op 3 Month	Post-Op 6 Month	Δ (6M–Pre-Op), 95% CI	*p*-Value	Effect Size (η^2^)
Central macula thickness (µm)	289.50 ± 16.26	282.50 ± 24.75	286.50 ± 16.26	290.00 ± 18.38	293.00 ± 19.80	+3.5 (−3.5 to 10.5)	0.190	0.04
Ganglion cell layer (µm)	22.50 ± 9.19	21.50 ± 14.85	21.50 ± 10.61	23.00 ± 11.31	22.00 ± 12.73	−0.5 (−5.0 to 4.0)	0.936	0.01
Inner plexiform layer (µm)	27.50 ± 9.19	24.50 ± 16.26	28.00 ± 11.31	26.50 ± 9.19	27.50 ± 10.61	0.0 (−4.5 to 4.5)	0.765	0.02
Peripapillary RNFL (µm)	67.11 ± 20.41	77.44 ± 17.07	71.78 ± 19.04	74.67 ± 20.49	77.80 ± 14.86	+10.7 (−3.5 to 25.0)	0.504	0.03

Abbreviations: Pre-op = preoperative; post-op = postoperative; RNFL = retinal nerve fiber layer thickness. Continuous variables are presented as mean ± standard deviation. Longitudinal changes were analyzed using repeated-measures ANOVA, and *p*-values reflect the overall effect of time. Mean differences (Δ) represent month 6 minus baseline values, with corresponding 95% confidence intervals. Effect sizes (partial eta squared, η^2^) reflect the overall effect of time. Values of 0.01, 0.06, and 0.14 indicate small, moderate, and large effects, respectively. Estimated minimum detectable within-subject differences (MDDs) were GCL 6.4 µm, IPL 6.4 µm, CMT 9.9 µm, and RNFL 20.2 µm.

**Table 4:** Longitudinal changes in optic nerve head parameters.

Parameter	Pre-Op	Post-Op 1 Week	Post-Op 1 Month	Post-Op 3 Months	Δ (3M–Pre-Op), 95% CI	*p*-Value	Effect Size (η^2^)
C/D area (mm^2^)	0.36 ± 0.25	0.39 ± 0.24	0.31 ± 0.25	0.39 ± 0.26	+0.03 (−0.06 to 0.12)	0.729	0.02
Rim area (mm^2^)	1.16 ± 0.48	1.03 ± 0.65	1.26 ± 0.38	1.03 ± 0.41	−0.13 (−0.46 to 0.20)	0.594	0.03
Disk area (mm^2^)	1.83 ± 0.26	1.80 ± 0.29	1.87 ± 0.26	1.90 ± 0.22	+0.07 (−0.06 to 0.20)	0.266	0.04
Cup volume (mm^3^)	0.18 ± 0.20	0.33 ± 0.49	0.14 ± 0.15	0.23 ± 0.26	+0.05 (−0.08 to 0.18)	0.583	0.03

Abbreviations: Pre-op = preoperative; post-op = postoperative; C/D = cup/disk; continuous variables are presented as mean ± standard deviation. Longitudinal changes were analyzed using repeated-measures ANOVA, and corresponding *p*-values reflect the overall effect of time. Mean differences (Δ) are expressed as month 3 minus baseline values, with corresponding 95% confidence intervals derived from paired comparisons. Effect sizes (partial eta squared, η^2^) reflect the overall effect of time across all measurement points. Values of 0.01, 0.06, and 0.14 indicate small, moderate, and large effects, respectively.

**Table 5:** Longitudinal choroidal thickness analysis.

Parameter	Pre-Op	Post-Op 1 Week	Post-Op 1 Month	Post-Op 3 Month	Post-Op 6 Month	Δ (6M–Pre-Op), 95% CI	*p*-Value	Effect Size (η^2^)
CTn (µm)	296.71 ± 31.03	293.71 ± 38.71	294.71 ± 33.63	294.29 ± 32.82	298.14 ± 33.03	+1.43 (−0.1 to 3.0)	0.059	0.06
CTf (µm)	308.43 ± 36.95	299.43 ± 32.95	298.14 ± 37.44	299.86 ± 35.05	301.43 ± 33.96	−7.00 (−18.0 to 4.0)	0.211	0.04
CTt (µm)	293.29 ± 22.42	289.43 ± 20.92	289.43 ± 23.40	289.86 ± 22.80	292.29 ± 23.16	−1.00 (−5.5 to 3.5)	0.197	0.03

Abbreviations: Pre-op = preoperative; post-op = postoperative; CTn = nasal choroidal thickness; CTf = subfoveal choroidal thickness; CTt = temporal choroidal thickness. Longitudinal changes were analyzed using repeated-measures ANOVA, and corresponding *p*-values reflect the overall effect of time. Mean differences (Δ) are expressed as month 6 minus baseline values, with corresponding 95% confidence intervals derived from paired comparisons. Effect sizes (partial eta squared, η^2^) reflect the overall effect of time across all measurement points. Values of 0.01, 0.06, and 0.14 indicate small, moderate, and large effects, respectively. The estimated minimum detectable within-subject difference (MDD) was 15.6 µm for subfoveal CT.

**Table 6:** Optical coherence tomography angiography (OCTA) parameters at baseline, and 1 and 3 months.

Parameter	Pre-Op	Post-Op 1 Month	Post-Op 3 Month	Δ (1M–Pre-Op), 95% CI	Δ (3M–Pre-Op), 95% CI	*p*-Value	FDR-Adjusted *p*-Value	Effect Size (η^2^)
Superficial Capillary Plexus								
Macula VD (%)	38.36 ± 7.30	34.92 ± 8.41	35.00 ± 4.41	−3.44 (−6.7 to −0.2)	−3.36 (−6.5 to 0.3)	0.009 * Pre-op-1M: 0.015	0.056	0.11
Macula SH VD (%)	38.74 ± 7.68	35.35 ± 8.97	34.18 ± 4.92	−3.39 (−7.0 to −0.3)	−4.56 (−8.8 to 0.8)	0.007 * Pre-op-1M: 0.011	0.056	0.10
Macula IH VD (%)	37.99 ± 6.97	34.34 ± 8.74	35.64 ± 5.24	−3.65 (−7.3 to −0.3)	−2.35 (−6.0 to 1.3)	0.020 * Pre-op-1M: 0.015	0.085	0.10
Foveal VD (%)	20.43 ± 10.63	13.32 ± 6.55	13.85 ± 5.29	−7.11 (−16.3 to 2.1)	−6.58 (−15.7 to 2.5)	0.368	0.535	0.04
Deep Capillary Plexus								
Macula VD (%)	38.35 ± 9.00	35.89 ± 8.66	34.47 ± 5.92	−2.46 (−6.7 to 1.8)	−3.88 (−8.4 to 0.6)	0.248	0.496	0.05
Macula SH VD (%)	38.68 ± 8.26	35.04 ± 7.21	35.10 ± 4.34	−3.64 (−8.1 to 0.8)	−3.58 (−8.0 to 0.9)	0.342	0.547	0.04
Macula IH VD (%)	37.44 ± 9.42	34.87 ± 8.79	35.22 ± 6.18	−2.57 (−7.2 to 2.1)	−2.22 (−6.7 to 2.2)	0.410	0.547	0.03
Foveal VD (%)	35.60 ± 13.54	20.94 ± 12.77	22.58 ± 12.88	−14.66 (−29.7 to 0.4)	−13.02 (−27.8 to 1.8)	0.055	0.176	0.06
FAZ and Flow Metrics								
FAZ (mm^2^)	0.278 ± 0.16	0.364 ± 0.13	0.372 ± 0.19	+0.086 (−0.08 to 0.25)	+0.094 (−0.09 to 0.27)	0.287	0.510	0.02
Choriocapillaris flow area (mm^2^)	1.91 ± 0.32	1.79 ± 0.23	1.82 ± 0.25	−0.12 (−0.34 to 0.10)	−0.09 (−0.31 to 0.13)	0.372	0.535	0.02
Retina flow area (mm^2^)	1.33 ± 0.52	1.44 ± 0.66	1.48 ± 0.13	+0.11 (−0.18 to 0.40)	+0.15 (−0.12 to 0.42)	0.890	0.890	0.01
Peripapillary Vessel Density								
Whole-image ppVD (%)	37.94 ± 7.54	36.17 ± 8.86	35.56 ± 7.43	−1.77 (−3.3 to −0.2)	−2.38 (−6.8 to 2.2)	0.016 * Pre-op-1M: 0.008	0.085	0.07
Inside disk ppVD (%)	35.94 ± 7.79	35.09 ± 8.87	38.72 ± 7.33	−0.85 (−3.5 to 1.8)	+2.78 (−0.3 to 5.8)	0.264	0.496	0.03
Peripapillary VD (%)	38.62 ± 11.41	36.90 ± 11.66	39.52 ± 11.13	−1.72 (−5.6 to 2.2)	+0.90 (−3.0 to 4.8)	0.435	0.547	0.02
SH ppVD (%)	38.61 ± 9.34	38.14 ± 11.21	34.34 ± 9.90	−0.47 (−3.9 to 3.0)	−4.27 (−9.3 to 0.8)	0.368	0.535	0.03
IH ppVD (%)	40.28 ± 10.08	38.23 ± 11.12	32.04 ± 15.54	−2.05 (−7.5 to 3.0)	−8.24 (−22.0 to 4.5)	0.121	0.323	0.05

Abbreviations: Pre-op = preoperative; post-op = postoperative; M = month; VD = vessel density; SH = superior hemisphere; IH = inferior hemisphere; FAZ = foveal avascular zone; ppVD = peripapillary vessel density. * Overall *p*-values refer to repeated-measures ANOVA results. To account for multiple comparisons across OCTA parameters, false discovery rate (FDR) correction was applied using the Benjamini–Hochberg procedure. Post hoc pairwise comparisons were performed across all timepoints using Bonferroni correction (adjusted significance threshold *p* < 0.0167); only pairwise comparisons significant before FDR correction are presented. Mean differences (Δ) represent the difference between each postoperative timepoint and baseline, with corresponding 95% confidence intervals derived from paired comparisons. Effect sizes (partial eta squared, η^2^) reflect the overall effect of time across all measurement points. Values of 0.01, 0.06, and 0.14 indicate small, moderate, and large effects, respectively. Estimated minimum detectable within-subject differences (MDDs) were superficial macular VD 4.6%, deep macular VD 6.0%, whole-image ppVD 2.2%, and FAZ 0.23 mm^2^.

## Data Availability

The data supporting the findings of this study are available from the corresponding author upon reasonable request. The data are not publicly available because they contain clinical information from human participants and are subject to institutional and ethical restrictions.

## Supplementary Materials

The following supporting information can be downloaded at https://www.mdpi.com/article/10.3390/diagnostics16183049/s1. Table S1: GATT indications and preoperative topical IOP-lowering medication burden according to baseline glaucoma severity; Table S2: Pearson’s correlation coefficients between choroidal thickness parameters and OCTA-derived metrics.
