# Supplementary material for: Evaluation of Posterior Segment Structural and Vascular Changes After Gonioscopy-Assisted Transluminal Trabeculotomy
Source: Diagnostics (Basel). 2026 Sep 20;16(18):3049. doi: 10.3390/diagnostics16183049 (PMC13605410; doi:10.3390/diagnostics16183049)
Supplement: Supplementary file 1 [file diagnostics-16-03049-s001.zip › Supplementary Table S1.pdf]

Supplementary Table S1. GATT indications and preoperative topical IOP-lowering medication burden according to baseline glaucoma severity

	Mild (n=17)	Moderate (n=9)	Severe (n=10)	Total (n=36)
**Indication for GATT**				
Uncontrolled glaucoma despite MTMT	1 (5.9%)	5 (55.6%)	6 (60.0%)	12 (33.3%)
Medication non-adherence	7 (41.2%)	2 (22.2%)	0 (0.0%)	9 (25.0%)
Medication side effects	3 (17.6%)	2 (22.2%)	2 (20.0%)	7 (19.4%)
Difficulty maintaining regular follow-up	6 (35.3%)	0 (0.0%)	2 (20.0%)	8 (22.2%)
	**Mild (n=10)**	**Moderate (n=7)**	**Severe (n=10)**	**Total (n=27)**
**Preoperative topical medication regimen**				
1 medication	1 (10.0%)	0 (0.0%)	0 (0.0%)	1 (3.7%)
2 medications	6 (60.0%)	1 (14.3%)	4 (40.0%)	11 (40.7%)
3 medications	3 (30.0%)	6 (85.7%)	6 (60.0%)	15 (55.6%)
Mean ± SD	2.20 ± 0.60	2.86 ± 0.35	2.60 ± 0.49	2.52 ± 0.58

Data are presented as n (%) unless otherwise indicated. Percentages for the primary surgical indication were calculated within the full baseline severity groups (mild, n=17; moderate, n=9; severe, n=10), and each eye was assigned one primary indication. MTMT: maximum tolerated medical therapy. Percentages are calculated within each severity group (column percentages). Preoperative topical treatment burden was reliably ascertainable in 27 eyes (mild, n=10; moderate, n=7; severe, n=10); percentages in this section were calculated using these available-case denominators. Treatment burden was calculated according to the number of active pharmacological classes, with each component of a fixed-combination medication counted as a separate class. The prescribed topical classes included prostaglandin analogues, β-blockers, carbonic anhydrase inhibitors, and α2-adrenergic agonists. Two eyes additionally received oral acetazolamide, which was not included in the topical medication count. GATT, gonioscopy-assisted transluminal trabeculotomy; IOP, intraocular pressure; MTMT, maximum tolerated medical therapy; SD, standard deviation.
