# Supplementary material for: Evaluation of Posterior Segment Structural and Vascular Changes After Gonioscopy-Assisted Transluminal Trabeculotomy
Source: Diagnostics (Basel). 2026 Sep 20;16(18):3049. doi: 10.3390/diagnostics16183049 (PMC13605410; doi:10.3390/diagnostics16183049)
Supplement: Supplementary file 1 [file diagnostics-16-03049-s001.zip › Supplementary Table S2.pdf]

Supplementary Table S2. Pearson's correlation coefficients between choroidal thickness parameters and OCTA-derived metrics.

	FAZ (r, p)	Superficial FVD (r, p)	Deep FVD (r, p)	Chorio-capillaris Flow (r, p)	Retina Flow Area (r, p)	Peripapillary VD (r, p)
**CTn**	–0.372, 0.411	0.225, 0.627	0.316, 0.490	0.021, 0.964	0.045, 0.924	–0.569, 0.183
**CTf**	–0.261, 0.572	0.205, 0.659	0.301, 0.512	0.070, 0.882	0.134, 0.775	–0.520, 0.231
**CTt**	–0.723, 0.066	0.506, 0.247	0.600, 0.154	0.335, 0.463	–0.157, 0.736	–0.352, 0.439

Abbreviations: CTn = nasal choroidal thickness; CTf = subfoveal choroidal thickness; CTt = temporal choroidal thickness; FAZ = foveal avascular zone; FVD = foveal vessel density; VD = vessel density. Statistical test: Pearson's correlation analysis.
